# Enhancing the growth, yield and physiological response of two lettuce (*Lactuca sativa* L.) cultivars through NFT system optimization

**DOI:** 10.3389/fpls.2025.1639002

**Published:** 2025-08-05

**Authors:** Paulo Pastor-Arbulú, Alfredo Rodríguez-Delfín

**Affiliations:** ^1^ Facultad de Ciencias, Centro de Investigación de Hidroponía y Nutrición Mineral, Universidad Nacional Agraria La Molina, Lima, Peru; ^2^ Centro de Investigación de Hidroponía y Nutrición Mineral, Universidad Nacional Agraria La Molina, Lima, Peru

**Keywords:** cash crop physiology, hydroponics and soilless culture, nutrient film technique (NFT), yield-contributing traits, sustainable agriculture

## Abstract

**Introduction:**

In the context of increasing pressure on agricultural resources, hydroponic systems such as the nutrient film technique (NFT) are gaining prominence for their ability to optimize water use and space efficiency, and crop productivity in controlled environments. Lettuce (*Lactuca sativa* L.), a high-value leafy vegetable, is a key cash crop in controlled-environment agriculture. Light quality and intensity -critical drivers of plant physiology- require constant monitoring in soilless systems to ensure consistent performance. However, the interaction effects of NFT system design and cultivar selection on physiological behavior and yield stability remain underexplored.

**Methods:**

This study evaluated the growth, yield, and physiological responses of two lettuce cultivars, Tropicana and Starfighter, cultivated in three NFT configurations: module I (8-channel) with a horizontal layout; and module II (13-channel) and module III (10-channel), both with pyramidal layouts. Although all the treatments were exposed to similar microenvironmental conditions, the photosynthetic photon flux density (PPFD) was monitored throughout the crop cycle to maintain light uniformity. Agronomic performance was evaluated through biometric parameters in roots, stems, leaves and heads, and the yield was calculated per unit area; while the physiological responses included measurements of relative and total chlorophyll content and nitrate reductase enzymatic activity.

**Results and discussion:**

Tropicana generally outperformed Starfighter, particularly in modules II and III, which also supported higher pigment accumulation and improved nitrogen metabolism across both cultivars. The highest yields were achieved by Tropicana in modules II (14.14 kg·m^-2^) and III (13.96 kg·m^-2^), closely followed by Starfighter in module II (13.45 kg·m^-2^). These findings highlight how strategic integration of system configuration and cultivar selection can increase physiological efficiency, stabilize yields, and promote sustainability in hydroponic lettuce production.

## 1 Introduction

In the coming decades, ensuring more food resources for an increasing population would be difficult, especially in urban zones, because of the backdrop of climate change, soil loss, and water scarcity. Therefore, sustainable food production is a pivotal challenge for humanity. As the global population grows and urbanization intensifies, food security strategies, through innovative agricultural practices and methods are crucial for addressing these issues, while contributing to environmental protection (Ahmed et al., 2021b). Soilless systems, such as hydroponics, aquaponics or aeroponics, have emerged as one of these solutions by minimizing land use, reducing water consumption, and enabling year-round crop production in urban areas (Vanacore et al., 2024).

Lettuce (*Lactuca sativa* L.) stands out for its significant economic importance among leafy vegetables, and it serves as a good model species for evaluating agronomic and physiological responses because it is representative of green leafy vegetables widely consumed worldwide and can be easily produced in various agricultural systems (Lei and Engeseth, 2021; Góis et al., 2024). Lettuce is the most cultivated cash crop in hydroponic systems, which consists of a soilless cultivation technology that applies nutrient solutions and artificial growing media (Ahmed et al., 2021a). This provides the ability to grow the plants in a shorter period, in poor soil quality areas, or in limited space all year round, regardless of the climate, since it can be grown in mesh houses or greenhouses, with more or less control of the microenvironmental conditions (Alneyadi et al., 2024; Vanacore et al., 2024).

The hydroponic cultivation techniques mostly consist of a static aeration system (floating root), the use of inert substrates with fertigation, or strategic irrigation techniques with a scheduled supply of nutrient solution by laminar flow, such as deep flow (DFT) and nutrient film (NFT). Among these systems, NFT is the most commonly used method for producing leafy vegetables and highly depends on electrical energy to recirculate the mineral solution (Góis et al., 2024). Various studies have shown that the NFT system promotes a relatively high growth rate, high biomass, and good uniformity in lettuce, an ideal model because of its short cycle, environmental sensitivity, and high commercial demand (Malca Soto, 2013; Suharjo and Suaib, 2022).

NFT is extensively utilized in the production of vegetables such as lettuce and consists of channels in which a constant flow of nutrient solution is maintained (Ahmed et al., 2021a; Alneyadi et al., 2024; Alkaabi et al., 2025). The net cups with plants were placed in these channels with the plant roots dangling in the thin film of the nutrient mixture. For the nutrient film technique system, PVC pipes with a diameter of 110 mm served as growth channels. The nutrient solution from the reservoir was pumped via a nutrient flow introduction mechanism into the growth channels, the ends of which were sealed with plastic end caps of the same diameter as those of the channels (Majid et al., 2020). The nutrient mixture is circulated and has a nutritional content that considers the needs of the plants and the root system grows on a mixture of micronutrients and macronutrients (Alkaabi et al., 2025).

Furthermore, several factors must be considered, such as when excessive water will occur and factors that decrease the amount of oxygen. The nutrient content of the NFT method is specially designed, with a maximum solution height of 3 mm, so that the water, nutrient, and oxygen needs can be met. This system allows plants to receive nutrients and water through circulation in shallow and sloped layers. It is also a planting technique that allows the development of lettuce on paragon pipes with a 1–5% slope. This system does not require growth media because the roots of plants are submerged in a thin layer of nutrients that circulate and are regulated via a timer (Suharjo and Suaib, 2022).

The overall health indicators of crops, such as their chlorophyll content, reflect their photosynthetic capacity, growth stage, and nitrogen status and are therefore highly important in precision agriculture. In addition, the activity of the enzyme nitrate reductase has been identified as a key indicator of physiological efficiency, as it regulates the conversion of nitrate into nitrite, a form assimilable by the plant, and is modulated by light, nitrogen availability, and phenological stage (Malca Soto, 2013; Chamoli et al., 2024). These responses reflect the ability of lettuce to maximize the use of solar energy, a determining factor in passive systems based on natural radiation (Valverde et al., 2009). The constant monitoring of these parameters is very important and informative for adjusting strategies to increase productivity by specialists since they are categorized as yield-contributing traits.

Hence, it is crucial to optimize the NFT designs and test them in different cultivars to provide scientific information to the entrepreneurial society that could develop effective solutions to current problems that involve urban food systems and evaluate the extent to which they help to increase productivity and stabilize yields in the context of resource scarcity and better use of the available space, regardless of the climate conditions or soil characteristics, as well as to provide an understanding of the physiology of the cash crop in these particular sustainable and affordable agricultural scenarios.

The present research aimed to compare the agronomic and ecophysiological behavior of the lettuce cultivars ‘Tropicana’ and ‘Starfighter’ developed in three adaptations (modules I, II and III) of the NFT hydroponic system to increase productivity together with microenvironmental condition management. This study considered the following measurements: typical growth parameters such as root length, plant height, and the fresh and dry weights of roots, stems, leaves, and heads; harvested yield; and key physiological indicators, including total and relative chlorophyll content and nitrate reductase enzymatic activity, which are related to light harnessing and nutrient assimilation efficiency.

## 2 Materials and methods

### 2.1 Microenvironmental conditions

The study was conducted at the Centro de Investigación de Hidroponía y Nutrición Mineral of the Universidad Nacional Agraria La Molina (-12.07982, -76.94774) in Lima, Peru, during the summer of 2021. To reduce high radiation and high temperatures, 50% shade mesh was used. The climatic conditions during the test were as follows: global radiation, 644 MJ·m^-2^·month^-1^; average PAR, 114.2 W·m^-2^ (522.89 μmol·m^-2^·s^-1^ PPFD); maximum and minimum temperatures, 31.5°C and 15.6°C, respectively; and 66.4% relative humidity and average day and night temperatures, 25.82°C and 20.43°C, respectively (**Supplementary Table S1**).

### 2.2 Plant material and growth conditions

‘Tropicana’ and ‘Starfighter’ lettuce seeds (curly type cultivars with heat tolerance), produced and commercialized by Johnny’s Selected Seeds (Winslow, Maine, USA), which were sown in a storage tray with a quarry sand substrate 0.5 to 1.00 mm in diameter, were used for screening and washing. After germination, the seedlings were watered with well water. After 8 days, the seedlings were selected for the first transplantation into plastic pots 4 cm in diameter, perforated in the center of the base, through which the roots were transferred; 12 days later, the seedlings and their respective pots were placed in the holes in the canals. For 25 days, the plants were nourished daily via the hydroponic nutrient film technique (NFT) system, which was configured according to Valverde et al. (2009) (Valverde et al., 2009) and Rodríguez-Delfín et al. (2022) (Rodríguez-Delfin et al., 2022). The nutrients of the plants were obtained from La Molina hydroponic solution (Valderrama et al., 2020), and the plants were stored in a 1,100 L capacity tank. The nutrient solution was recirculated via a 372.85 W electric pump, which fed the plants of the pyramid and horizontal modules.

### 2.3 Experimental design and NFT configurations

Sixty lettuce plants were sampled 48 days after germination, at which point they reached optimal development and were subsequently harvested. A completely randomized design with a 2 × 3 factorial arrangement was used, corresponding to two lettuce cultivars (Tropicana and Starfighter) and three NFT modules, and a total of 6 treatments with 10 replications each were evaluated. The nutrient film technique (NFT) hydroponic system used in this study consisted of three distinct modules with varying configurations. Module I featured a horizontal layout comprising eight channels, each with a diameter of 7.5 cm and a length of 3 m, arranged within a structure 1.5 m wide (**Supplementary Figures S1A-B**). Module II employed a pyramidal configuration with 13 channels of the same dimensions (7.5 cm diameter, 3 m length) and a width of 1.5 m (**Supplementary Figures S1C-E**). Similarly, Module III followed a pyramidal design but included 10 channels, measuring 7.5 cm in diameter and 3 m in length, within a 1.5 m-wide framework (**Supplementary Figures S1F-H**).

### 2.4 Determination of growth and yield responses

The agronomic variables evaluated included plant height; root length; and fresh and dry weights of the roots, stems, leaves and head. The samples were placed in a temperature-controlled oven at 70°C for 48 hours. The yield was calculated from the total amount of lettuce harvested according to the accumulated fresh weight of heads produced per unit area, and each module occupied an area of 4.5 m^2^. A total of 120, 190 and 150 lettuce plants are harvested in modules I, II and III, respectively (Rodríguez-Delfin et al., 2022).

### 2.5 Determination of ecophysiological responses

At the physiological level, the total chlorophyll content was measured via spectrophotometry via the methods of Lichtenthaler et al. (1983) (Lichtenthaler and Wellburn, 1983) and Wellburn (1994) (Wellburn, 1994), with some modifications; the SPAD index, which automatically calculates the relative chlorophyll content present in the leaf, was obtained via the SPAD 502-Plus portable meter; and the activity of the enzyme nitrate reductase in young leaves was quantified via the method of Neyra et al. (1974) (Neyra and Hageman, 1974).

### 2.6 PPFD monitoring in time

With the use of a Quantum Light Meter, the light intensity was monitored over time from the first week after the second transplant, over a period of 18 days, with a total of 6 measurements (every 3 days). The solar control PAR values (in the open air and at noon) and under the green mesh with 50% shade (each module is divided into 9 quadrants, and its respective value is recorded) were recorded, all in PPFD units (μmol·m^-2^·s^-1^) (**Supplementary Figure S4**).

### 2.7 Statistical analysis

The data were analyzed via factorial ANOVA and the Tukey´s honest significant difference (HSD) test (p < 0.05) after verification of normality and homogeneity of variance. Pearson correlation coefficient was also calculated between the ecophysiological and photoabsorption variables. All analyses were run with the Minitab 19 or Python programs.

## 3 Results

### 3.1 Development of the lettuce cultivars in three different NFT configurations

**Figure 1:**
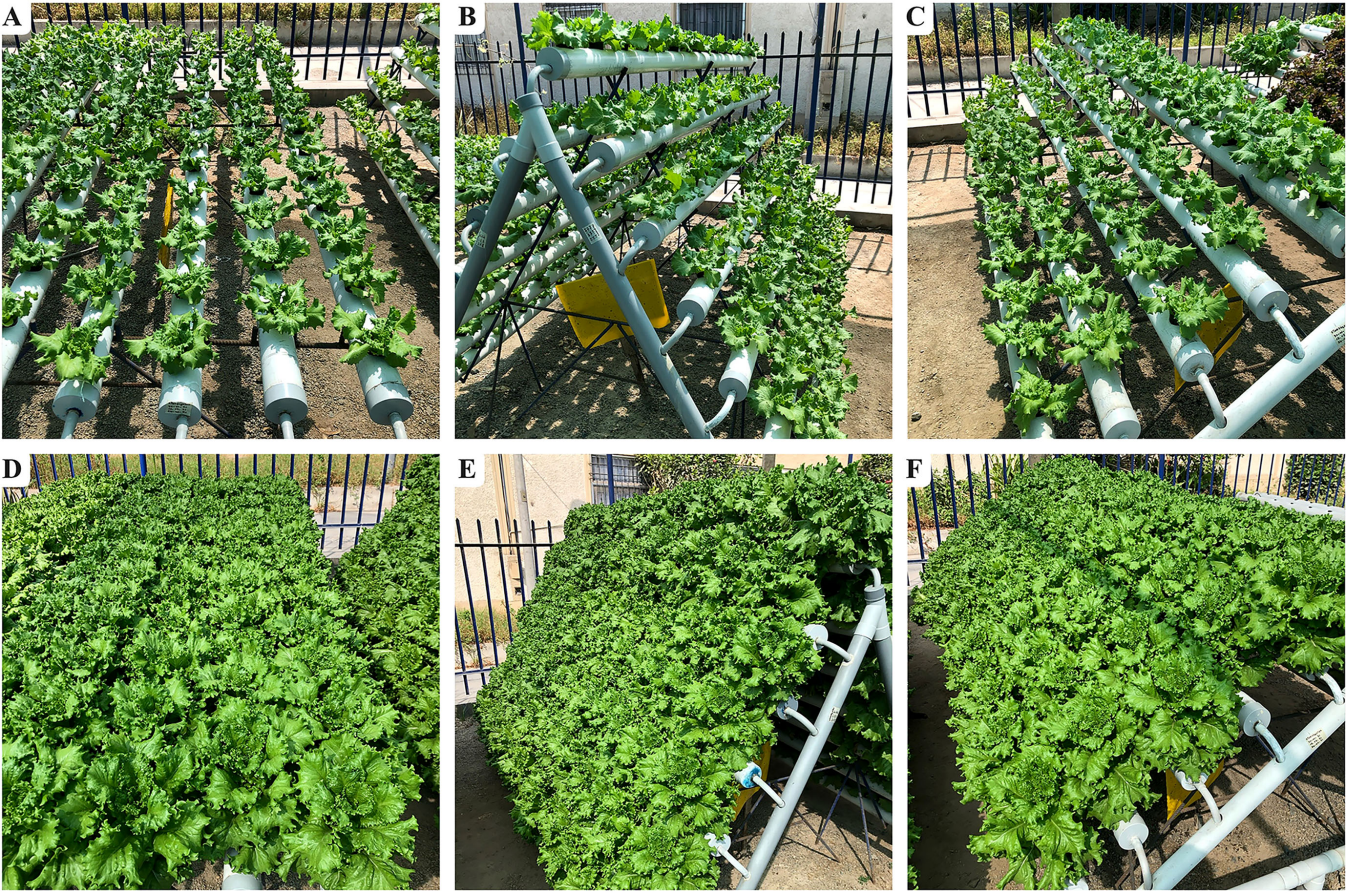
Lettuce cultivars grown in three hydroponic NFT types. 30 days after germination: module I **(A)**, module II **(B)**, module III **(C)**; 45 days after germination: module I **(D)**, module II **(E)**, module III **(F)**.

**Figures 1** shows the lettuce plants of ‘Tropicana’ and ‘Starfighter’ that started developing in NFT modules I, II and III 20 days after germination of the seeds. The plants were almost ready for harvesting after 25 days of mineral solution supply and recirculation. At the macroscopic level, there were no evident differences between the treatments, and the lettuce plants demonstrated an overall healthy state, which is why it is crucial to collect further data on key biometric and physiological parameters. Moreover, there is notorious leaf expansion and development and overall differences in size within just 15 days of NFT cultivation between the pictures illustrating each module.

### 3.2 Growth and yield responses

#### 3.2.1 Fresh and dry biomass of plant organs and harvested yield

**Figure 2:**
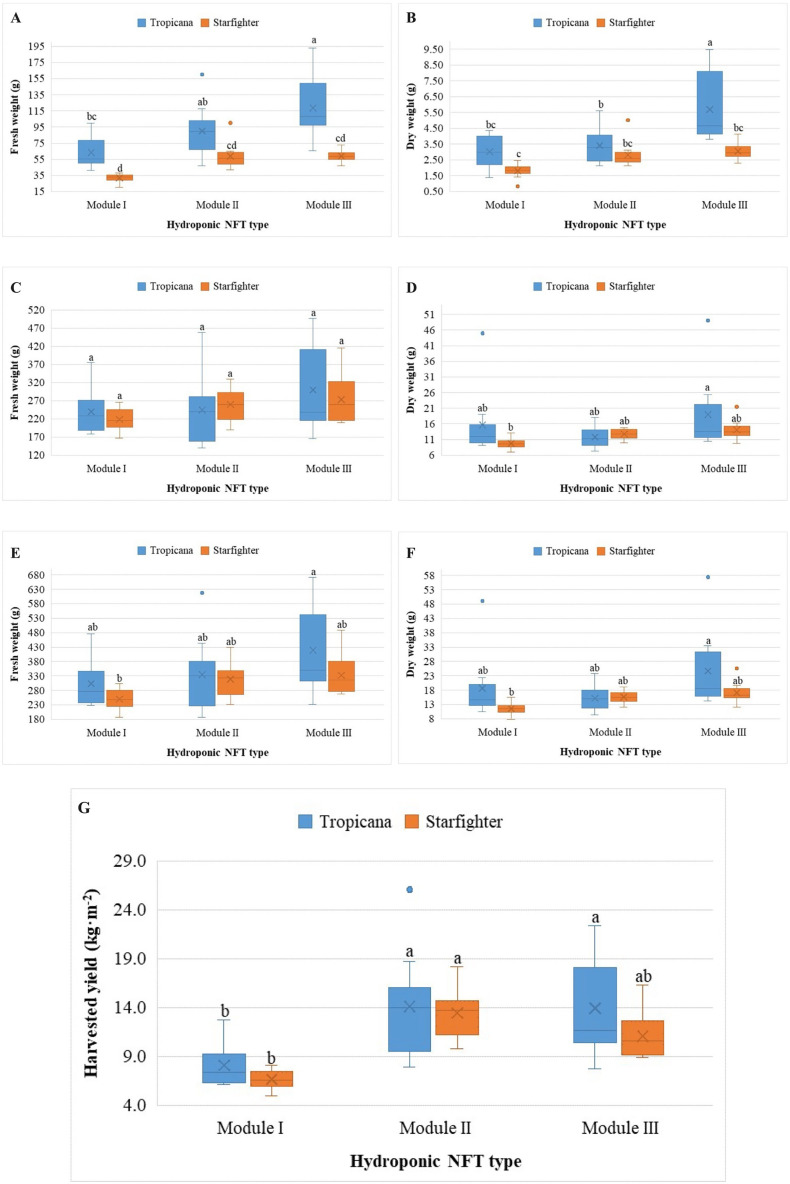
Stems: fresh **(A)** and dry **(B)** weights; leaves: fresh **(C)** and dry **(D)** weights; heads: fresh **(E)** and dry **(F)** weights; harvested yield **(G)** of the Tropicana and Starfighter cultivars, developed in three NFT modules. Lowercase letters correspond to the Tukey´s HSD test (p<0.05) for multiple comparisons after the factorial ANOVA. Treatments that do not share a letter have significantly different means.

Among the agronomic variables evaluated, fresh and dry root biomass, as well as length, did not significantly differ between treatments (p > 0.05), indicating that root development is independent of cultivar, as is the type of NFT system (**Supplementary Figure S2**). In contrast, significant differences were found in the fresh and dry biomasses of the stems, both for the effects of modulus, cultivar and the interaction between both factors (p < 0.02). Modules II and III favor greater stem biomass, while greater development of the stems of the Tropicana cultivar than those of the Starfighter cultivar is evident (**Figures 2A, B**).

The analysis of the fresh and dry weights of the leaves revealed a mixed behavior; on the one hand, in the FW (**Figure 2C**), there were no significant differences between the treatments (p > 0.05). However, considering the DW (**Figure 2D**), the record of Tropicana in module III was significantly greater than that of the Starfighter in module I (p < 0.05). On the other hand, the analysis of fresh (**Figure 2E**) and dry (**Figure 2F**) head weights revealed significant differences between the treatments (p < 0.05); mainly, the registration of Tropicana in module III was significantly greater than that of the Starfighter in module I, and the remaining treatments exhibited similar behavior.

In relation to yield (**Figure 2G**), significant differences were observed between treatments, particularly between the interaction of the module and the cultivar (p < 0.05). Module II and the Tropicana cultivar in module III presented an average yield that was statistically greater than that of the other combinations, especially with respect to the yield of module I.

#### 3.2.2 Plant height

**Figure 3:**
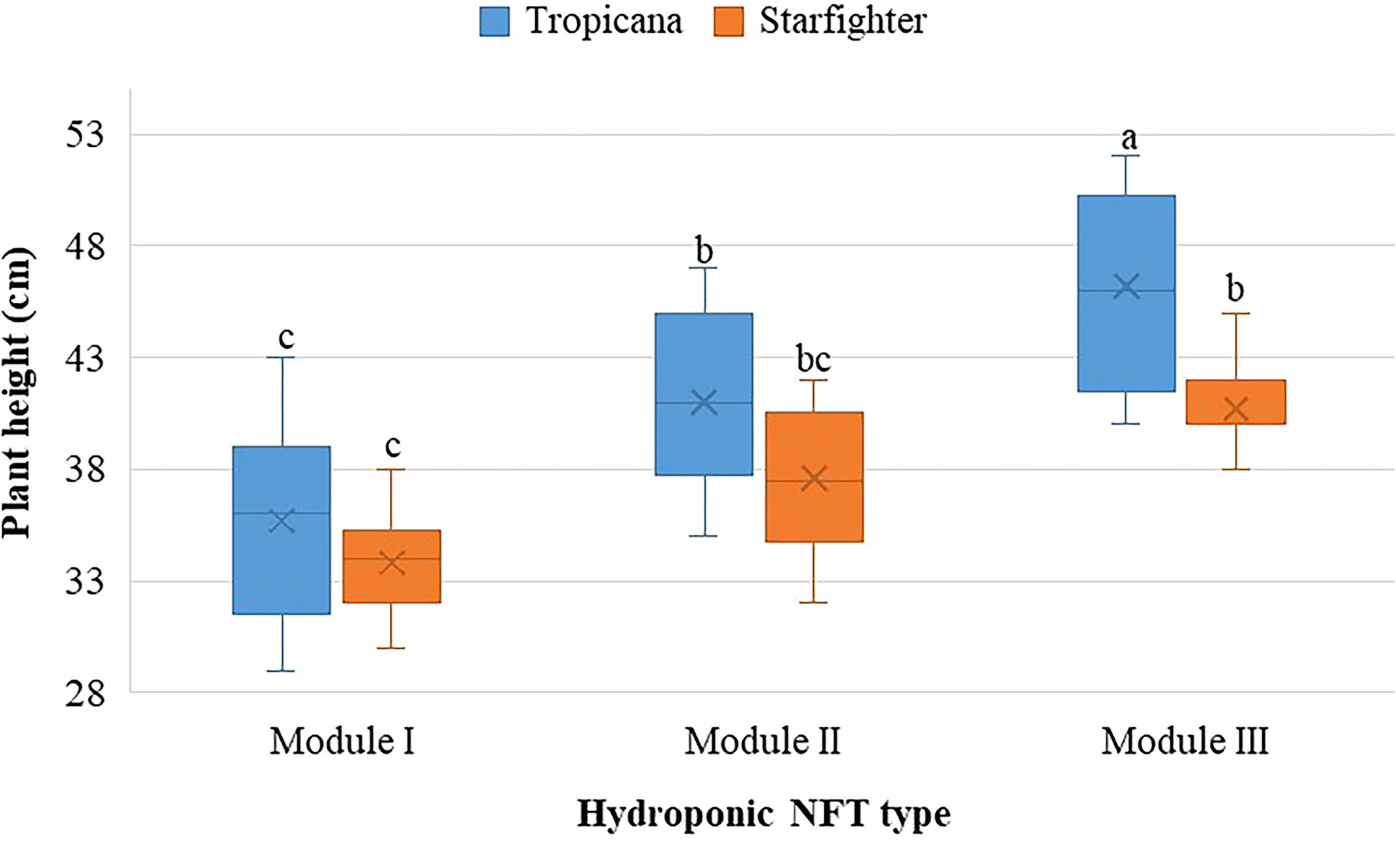
Height of lettuce plants of the Tropicana and Starfighter cultivars, developed in three NFT modules. Lowercase letters correspond to the Tukey´s HSD test (p<0.05) for multiple comparisons after the factorial ANOVA. Treatments that do not share a letter have significantly different means.

With respect to plant height (**Figure 3**), significant differences were observed between treatments (p < 0.05), indicating that both the variety and the type of modulus affect the growth of lettuce. The Tropicana cultivar in module III clearly achieved greater elongation. For module II, both varieties showed similar behavior, similar to that of the Starfighter in module III. On the other hand, module I generated smaller plants.

### 3.3 Physiological responses

**Figure 4:**
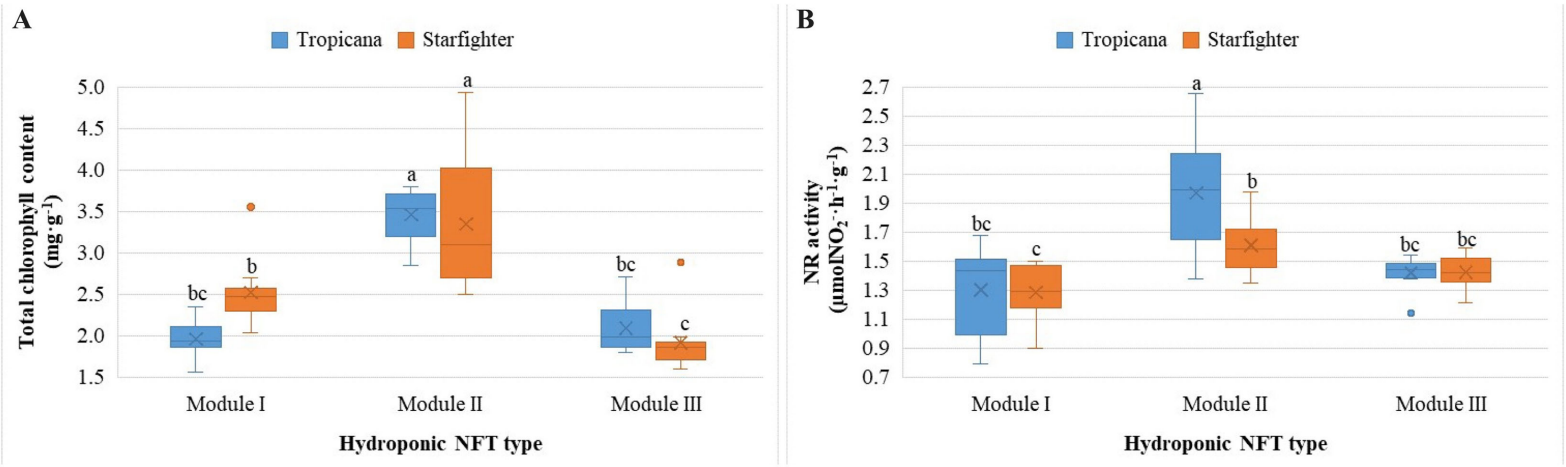
Total chlorophyll content **(A)** and the nitrate reductase (NR) enzymatic activity **(B)** in the leaves of lettuce plants of the Tropicana and Starfighter cultivars, developed in three NFT modules. Lowercase letters correspond to the Tukey´s HSD test (p<0.05) for multiple comparisons after the factorial ANOVA. Treatments that do not share a letter have significantly different means.

The total chlorophyll content (**Figure 4A**) significantly differed among the treatments (p < 0.05), especially between the records of module II and those of the other modules, regardless of the cultivar (p > 0.4). Moreover, the photosynthetic health of Starfighter plants was better in module I than in module III. In contrast, the SPAD index (**Supplementary Figure S3**) did not significantly differ (p > 0.3) between the treatments. In general, differences are observed in terms of photoabsorption patterns and assimilation of nutrients.

The activity of the nitrate reductase (NR) enzyme (**Figure 4B**) was significantly affected by both factors (p < 0.05), suggesting that the joint modulation of this enzyme, which is key in nitrogen metabolism, depends on the NFT system and the cultivar. Compared with the other modules, Tropicana in module II clearly registered a greater rate of reduction of nitrates to nitrites but also Starfighter with its counterpart in module I. The cultivars in module III behaved similarly. Differences are also observed in terms of photoabsorption patterns and assimilation of nutrients.

**Figure 5:**
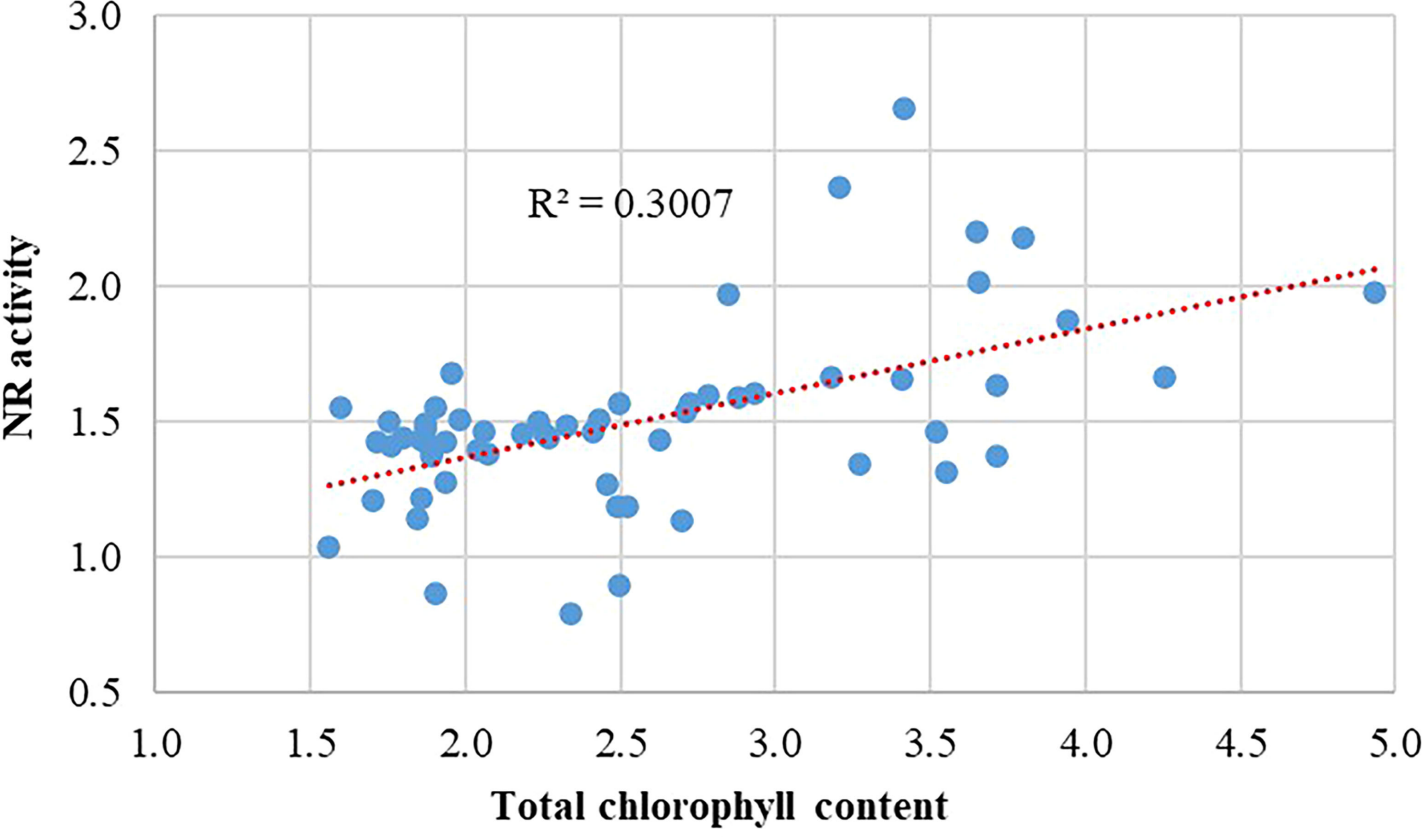
Pearson correlation between total chlorophyll content and the nitrate reductase (NR) enzymatic activity in the leaves of lettuce plants of the Tropicana and Starfighter cultivars, developed in three NFT modules.

In addition, the Pearson correlation between the analyzed ecophysiological variables was highly significant (r = 0.548; p < 0.01) (**Figure 5**).

Agronomically, these results show that, compared with module II, module I produces smaller lettuce plants, Tropicana produces more elongated plants in module III, and the choice of module is indistinct for Starfighter plants. The fresh leaf biomass was independent of the cultivar or module; however, greater dry matter production was observed in Tropicana leaves in module III than in those in the Starfighter leaves in module I. Regarding the ecophysiological and photoabsorption variables, first, with respect to the total chlorophyll content, module II achieved better results, regardless of the cultivar. For the quantification of NR enzymatic activity, module II again produced the best results, highlighting the Tropicana cultivar. These parameters are directly correlated with nutrient assimilation (Kanash et al., 2023; Liu et al., 2024).

## 4 Discussion

Significant interactions between the cultivar and the type of module were revealed. In terms of vegetative growth, the Tropicana cultivar presented greater plant height and head dry weight than did the Starfighter cultivar did, particularly in module III. This superiority could be related to a higher conversion efficiency of photosynthetically active radiation (PAR), even when the average values of light intensity were similar between the modules (~558–580 μmol·m^-2^·s^-1^). This finding coincides with previous studies that revealed a strong varietal influence on the efficiency of the use of light and nutrient assimilation in the NFT system, as reported by Espinoza et al. (2019) (Espinoza et al., 2019) and Valverde et al. (2009) (Valverde et al., 2009).

The highest values of fresh and dry weights of leaves and heads were also observed for Tropicana in module III, suggesting better adaptation of this cultivar to the configuration of the system. This pattern is consistent with what was reported by De M. N. Góis et al. (2024) (Góis et al., 2024), who reported that performance in the NFT system improved significantly under conditions of precise management of EC and pH, which are parameters that were controlled in all the modules evaluated. The highest yields were obtained in module II, regardless of the cultivar, although Tropicana also generated a similar harvest in module III. The differences with respect to module I are mainly explained by the lower density of plants, although the cultivars developed in this module, in general, also presented lower leaf and head fresh weights. These results are partially consistent with what was reported by Rodríguez-Delfín et al. (2022) (Rodríguez-Delfin et al., 2022), where the highest average yield was recorded in a 10-carcass system (similar to module III) in lettuce plants of the Ariana variety. However, the 8- and 13-channel systems showed no significant differences. Previously, Rodríguez-Delfín et al. (2001) (Rodríguez-Delfín et al., 2001) reported that different lettuce cultivars produce varied yields in deep flow technique (DFT) systems, which allow up to 27 plants per m^2^, with maximums between 2.74 and 3.38 kg·m^-2^, which is much lower than the maximums recorded in this study of 11.07 to 14.14 kg·m^-2^. In addition to pointing to a greater efficiency of NFT-modified systems than of DFT-modified systems, these results show that important differences in phenotype factors are expected in this species.

Similarly, Chamoli et al. (2024) (Chamoli et al., 2024) and Suharjo and Suaib (2022) (Suharjo and Suaib, 2022) highlighted that the structural design of the NFT system (including the gutter slope, substrate type, and module orientation) significantly influences light distribution and root oxygenation, which could explain the observed differences in growth, yield and physiological responses. This is also supported by the findings of Rodríguez-Delfín et al. (2001) (Rodríguez-Delfín et al., 2001) and (2022) (Rodríguez-Delfin et al., 2022), who reported significant differences between the configurations of the DFT and NFT modules, respectively.

The total chlorophyll values were greater in both cultivars within module II, where the highest activity of the nitrate reductase (NR) enzyme was also recorded. This correlation suggests that higher light intensity, combined with a module architecture that favors a homogeneous distribution of light, favors nitrogen assimilation, which is consistent with what was stated by Gupta (2020), who described the light activation of NR as a key step in the nitrate-to-nitrite reduction pathway. In contrast, the SPAD index did not significantly differ, which could be explained by its nature as an indirect estimator of relative chlorophyll content, which is less sensitive to subtle changes in photosynthetic activity. These results are consistent with those of Rodríguez-Delfín et al. (2022) (Rodríguez-Delfin et al., 2022), who reported that SPAD index measurements did not significantly differ between modules. Taha et al. (2024) (Taha et al., 2024) reported a strong correlation between the total chlorophyll content and SPAD units, which is well supported by background scientific evidence that the SPAD index is a reliable proxy for monitoring the stability of photosynthetically active pigments in lettuce plants. However, the methods described that the sampled leaves were exactly the same, in contrast with this study, where a random sampling of the leaves was performed, which could explain the differences between these two methods of quantification in terms of both total and relative chlorophyll contents.

In addition, studies such as those by Soto (2013) (Malca Soto, 2013) and Pinto et al. (2014) (Pinto et al., 2014) confirmed that high levels of nitrogen in nutrient solution significantly increase NR activity, generating a more efficient environment for amino acid and protein synthesis. However, in this experiment, the differences in NR activity between modules seem to be more associated with the photosynthetic efficiency between cultivars and the design of each NFT system, which might favor better light harnessing by the lettuce plants than with the concentration of nutrients, since this was constant in all the treatments. From a metabolic perspective, the high levels of NR activity found, especially in the Tropicana cultivar in module II, suggest a relatively high efficiency of nitrogen assimilation, which translates into a relatively low risk of nitrate accumulation in edible tissues (Coronel et al., 2009). This is an increasingly relevant quality criterion, as emphasized by Valverde et al. (2009) (Valverde et al., 2009) and Majid et al. (2020) (Majid et al., 2020), who related low nitrate accumulation to efficient recirculation and light control techniques.

In environments with natural shadows, irradiation/photon fluxes in the blue (400 to 500 nm) and red (600 to 700 nm) regions are relatively small, whereas photon fluxes in the green (500 to 600 nm) and far red (700 to 750 nm) regions are relatively enriched (Kusuma et al., 2021). This situation is expected to be replicated by means of a green mesh with 50% shade; in lettuce, significant increases in leaf expansion and leaf area have been reported, especially in tolerant cultivars. Shade tolerance is defined as an increase in leaf area in response to shade-like light, and shade avoidance is defined as an increase in stem elongation (Kusuma et al., 2021). In this respect, the Tropicana cultivar showed a greater tendency to elongate. In contrast, Starfighter generated more compact plants. In module I, taller plants with fresh leaf weights similar to those of their counterparts were recorded. In modules II and III, the lettuce with the largest size was observed. In general, the Starfighter cultivar presented less fresh and dry stem biomass, so it is possible to infer that it is a tolerant cultivar whose leaf area tends to increase. In contrast, Tropicana comparatively increased these parameters by 80% and can be categorized as a cultivar that avoids shade. This variable also explains some of the significant differences between fresh and dried head weights.

A limiting factor for photosynthetic organisms is their light-harvesting efficiency, i.e., the efficiency of their conversion of light energy into chemical energy (Chen and Blankenship, 2011). This is why it is important to secure and maintain light quality under microenvironment conditions. As shown in **Appendix A**, the photosynthetic photon flux density (PPFD) was monitored in time to compare with the public irradiance data of the zone, which was generally similar to the practical registration of photosynthetically active radiation (PAR), which is key in the quantum efficiency of plants, and it slightly varied between modules, with module II presenting the highest average (580.76 μmol·m^-2^·s^-1^). In a mesh house, microenvironmental conditions (such as relative humidity, temperature and irradiance) cannot be completely controlled and depend on the climate conditions in a specific geographic zone; however, as explained previously, a 50% shade mesh could benefit leafy vegetables used as a light management strategy to increase their productivity and yield. The scientific literature emphasizes that a moderate and stable PPFD (400–600 μmol·m^-2^·s^-1^) optimizes the rate of photosynthesis without inducing photoinhibition, as noted by Valverde et al. (2009) (Valverde et al., 2009), Kusuma et al. (2021) (Kusuma et al., 2021) and Zhen et al. (2022) (Zhen et al., 2022). However, the findings of Pennisi et al. (2020) (Pennisi et al., 2020) suggest that, to optimize yield in lettuce, it would be better to grow plants with a light intensity of 250–300 μmol·m^-2^·s^-1^.

In terms of sustainability, the NFT systems analyzed were shown to be highly efficient in the use of resources, especially water and nutrients, which has been widely documented in studies such as those by Barbosa et al. (2015) (Barbosa et al., 2015) and Maestre-Valero et al. (2018) (Maestre-Valero et al., 2018), who reported savings of up to 90% in water consumption compared with conventional agriculture. These findings are also consistent with those of Majid et al. (2020) (Majid et al., 2020), Chamoli et al. (2024) (Chamoli et al., 2024) and Vanacore et al. (2024) (Vanacore et al., 2024), where the multiple advantages of water use efficiency and space optimization for profitable purposes by soilless cultivation provide to the farmer community, complemented by the results presented in this research in relation to high yield stability (11–14 kg of fresh lettuce heads per square meter), with physiologically healthy plants at the macroscopic (key for consumers) and biochemical levels, independent of cultivar selection and with the potential of scalability to be a solution for microeconomics in urban food systems of important leafy vegetable cash crops, such as lettuce, spinach and basil. Finally, further research is needed that combines light efficiency management strategies and the collection of photoabsorption data with the design of NFT modules II or III to explore ways to increase both yield and productivity in sustainable lettuce cultivation.

## 5 Conclusions

Both the choice of lettuce cultivar and the configuration of the hydroponic NFT type play critical roles in determining growth performance, yield, and key physiological responses. The Tropicana cultivar generally outperformed the Starfighter cultivar in terms of fresh and dry biomass accumulation, particularly in modules II and III. These same configurations also promoted increased total chlorophyll content and nitrate reductase enzymatic activity. While all the modules were maintained under uniform environmental conditions, light intensity was monitored throughout the crop cycle to ensure consistent exposure. The combination of the Tropicana cultivar with modules II or III was the most effective, maximizing biomass production and favoring physiological parameters directly linked to crop quality and yield stability. These findings provide valuable insights for optimizing NFT configurations on the basis of cultivar selection and microclimatic conditions, contributing to the development of highly productive, resource-efficient, and scalable strategies for a leafy cash crop development in controlled-environment soilless systems in order to increase sustainable and high-efficiency production, effectively competing with conventional agriculture.

## Data Availability

The original contributions presented in the study are included in the article/**Supplementary Material**. Further inquiries can be directed to the corresponding author.

## Appendices

Appendix A: Irradiance, PAR and PPFD monitoring in time.

**Supplementary Figure S4** shows the irradiance data recorded by the NASA POWER Data Access Viewer, with average values of 21.47 MJ·m^-2^·day^-1^ of global radiation and 9.87 MJ·m^2^·day^-1^ PAR (**Supplementary Figure S4A**). Next, the behavior of the PAR is observed specifically for each module; each color curve represents one of the 9 quadrants, and the red curve corresponds to the control in the open air and at noon (943.67 μmol·m^-2^·s^-1^); module I (558 μmol·m^-2^·s^−1^) (**Supplementary Figure S4B**); module II (580.76 μmol·m^-2^·s^-1^) (**Supplementary Figure S4C**); and module III (558.82 μmol·m^-2^·s^-1^) (**Supplementary Figure S4D**).
