# Supplementary material for: Enhancing the growth, yield and physiological response of two lettuce (Lactuca sativa L.) cultivars through NFT system optimization
Source: Front Plant Sci. 2025 Aug 5;16:1639002. doi: 10.3389/fpls.2025.1639002 (PMC12361213; doi:10.3389/fpls.2025.1639002)
Supplement: Supplementary file 1 [file SupplementaryFile1.zip › Supplementary Material/Table 3.DOCX]

Supplementary Material

# Supplementary Figures


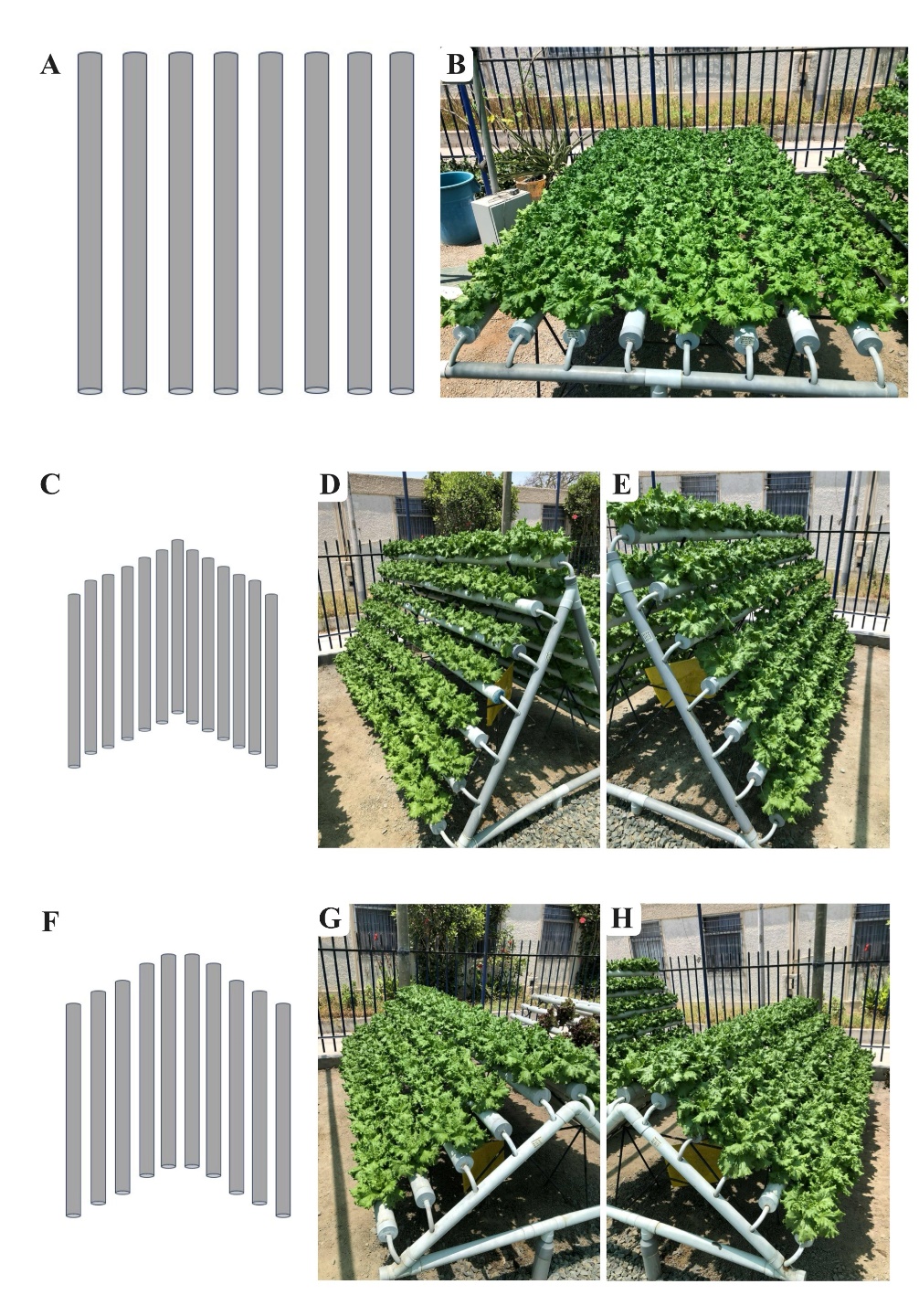


**Supplementary Figure 1**. Hydroponic NFT types. Modules I: perpendicular view **(A)** and front picture **(B)**. Module II: perpendicular view **(C)**, left-side picture **(D)** and right-side picture **(E)**. Module III: perpendicular view **(F)**, left-side picture **(G)** and right-side picture **(H)**.

**B**

**A**


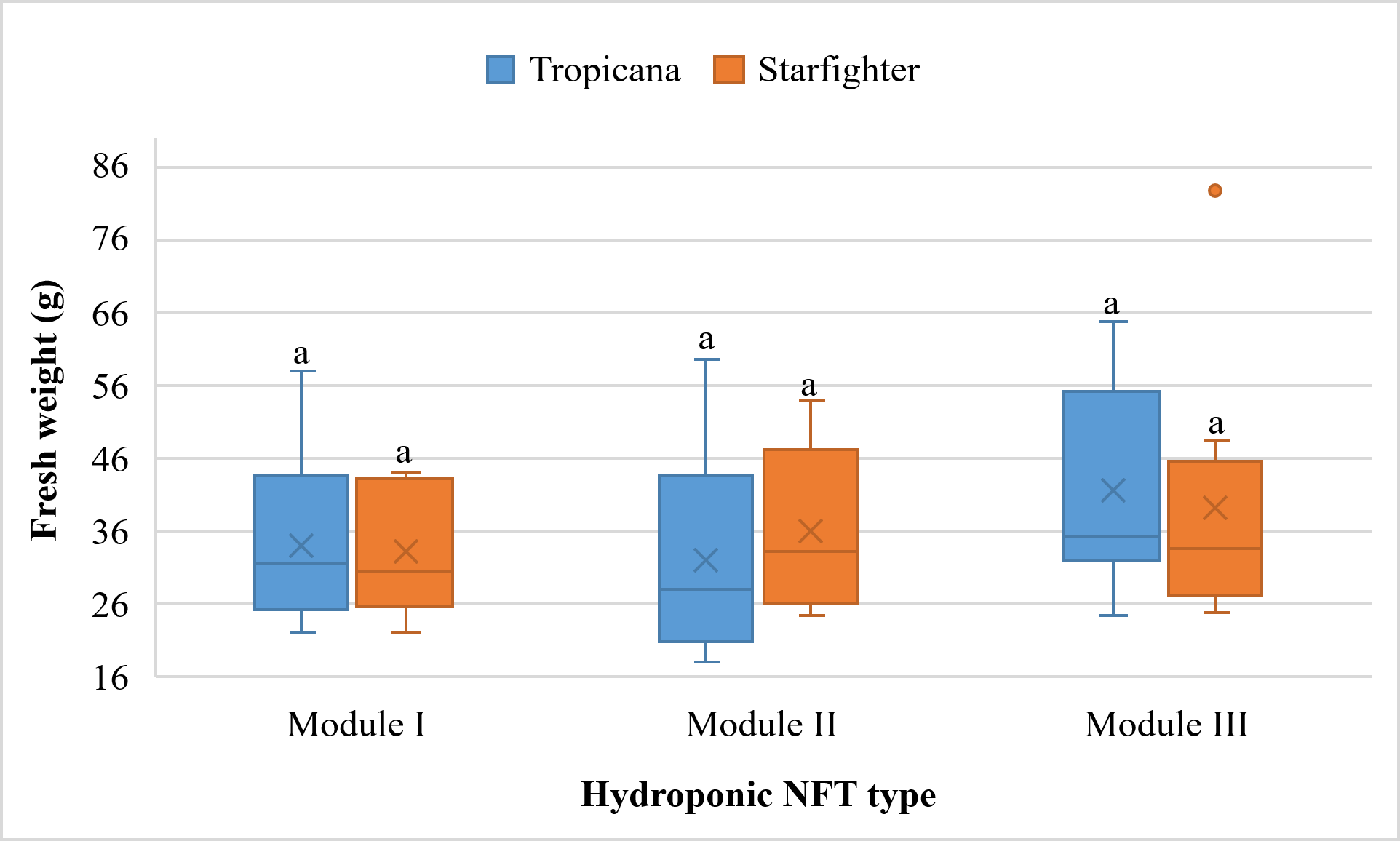

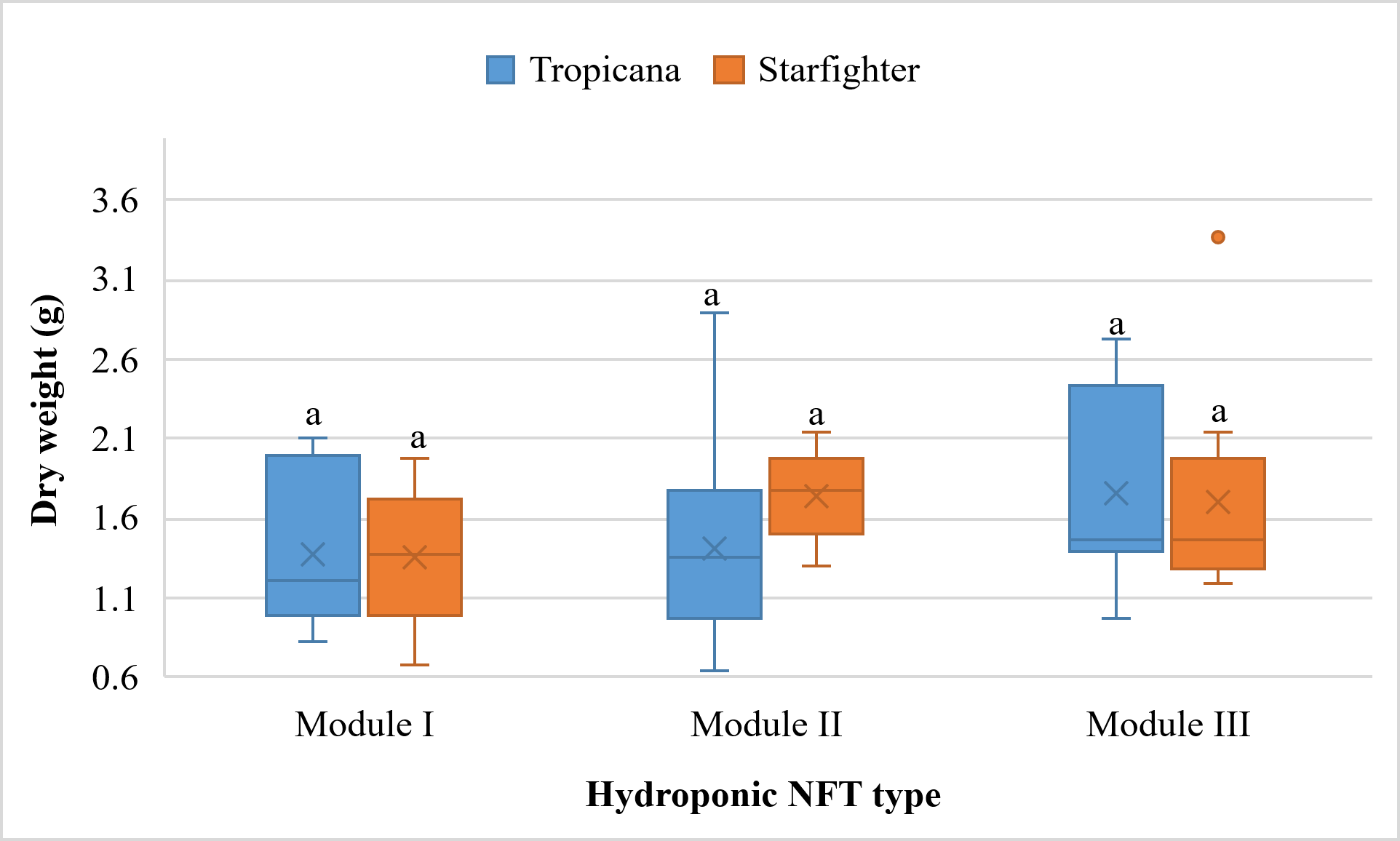


**C**


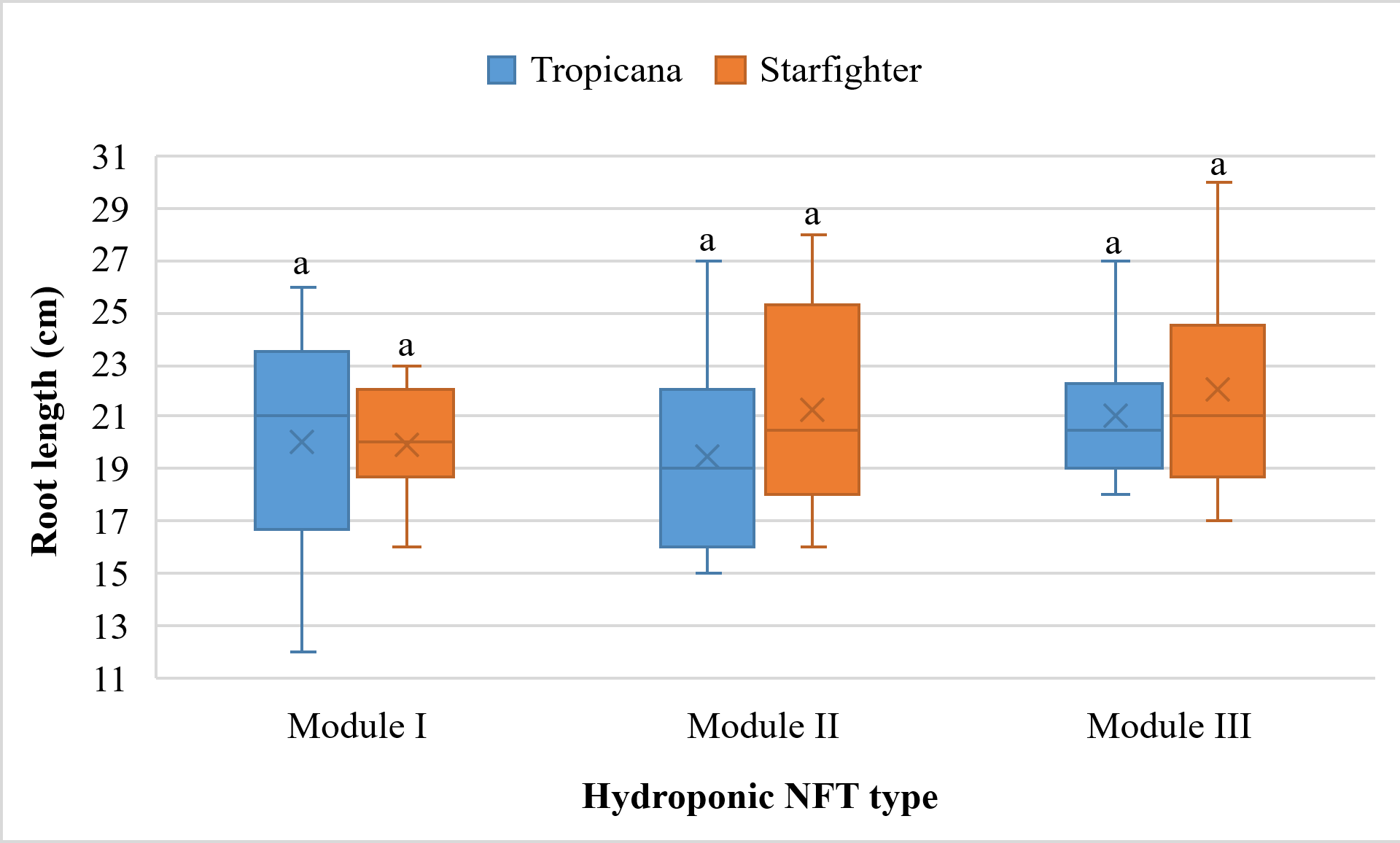


**Supplementary Figure 2.** Fresh **(A)** and dry **(B)** root weights; root length **(C)** of lettuce plants of the Tropicana and Starfighter cultivars, developed in three NFT modules.

**
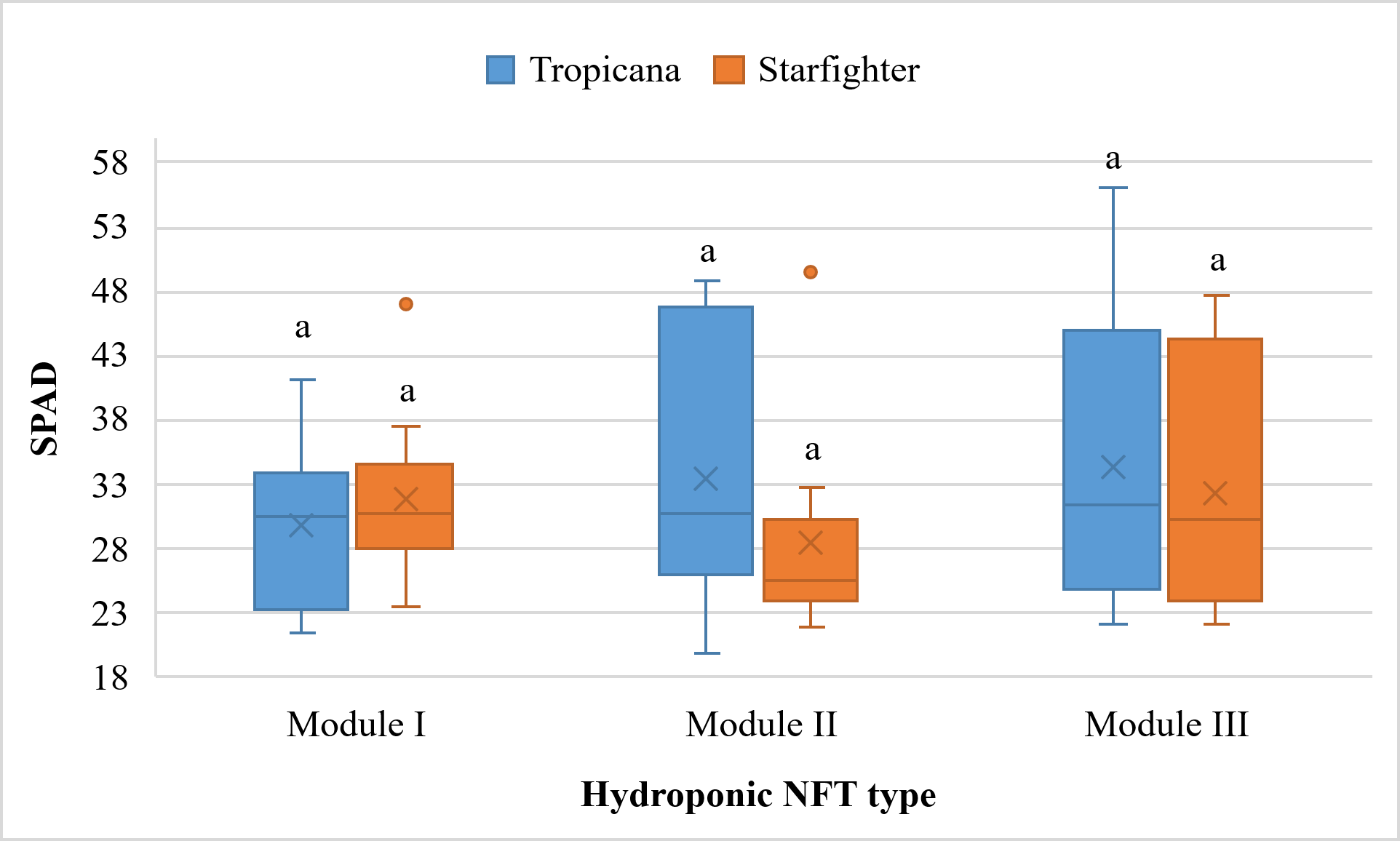
**

**Supplementary Figure 3.** SPAD index of lettuce plants of the Tropicana and Starfighter cultivars, developed in three NFT modules.

**B**

**A**


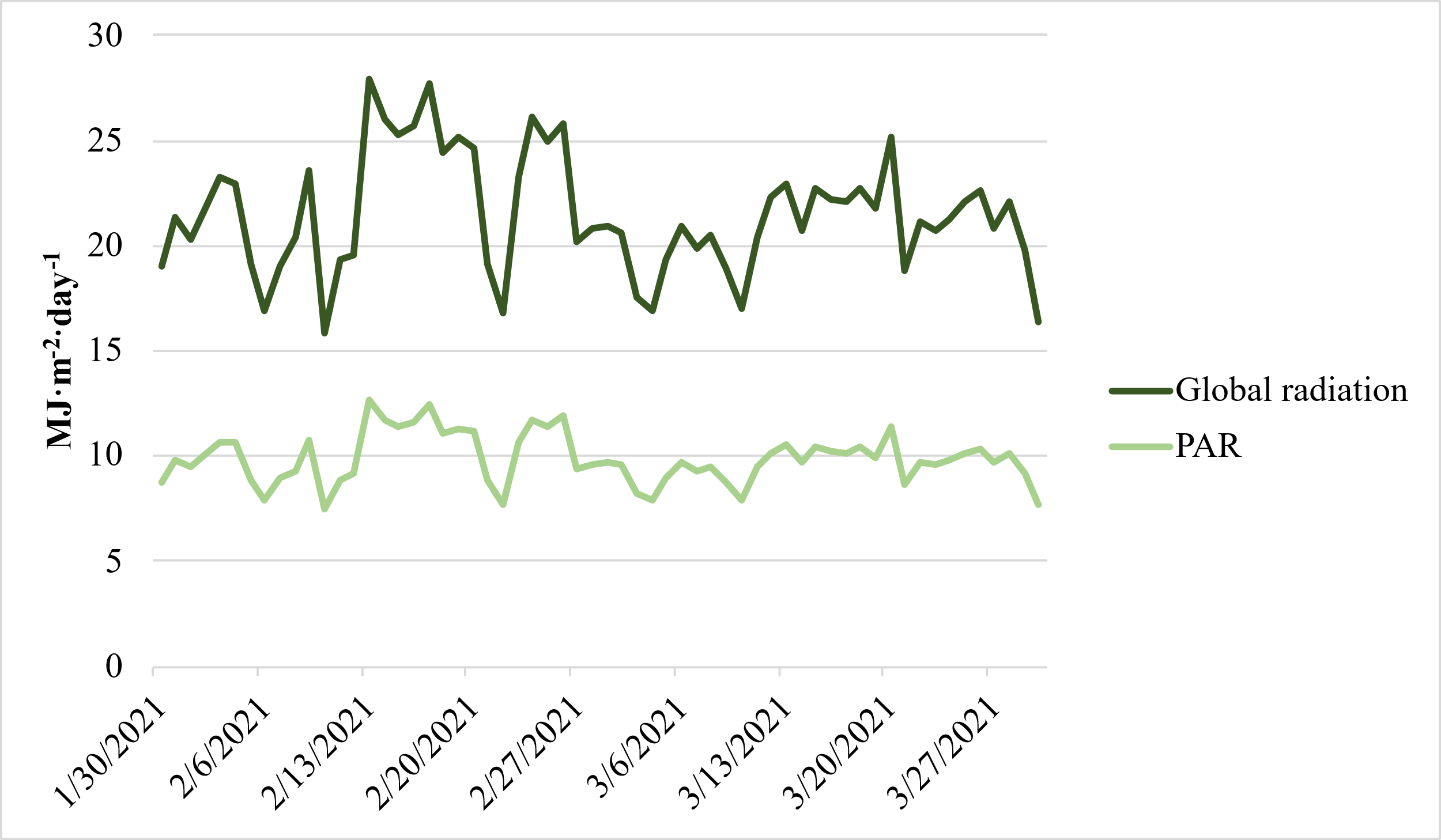

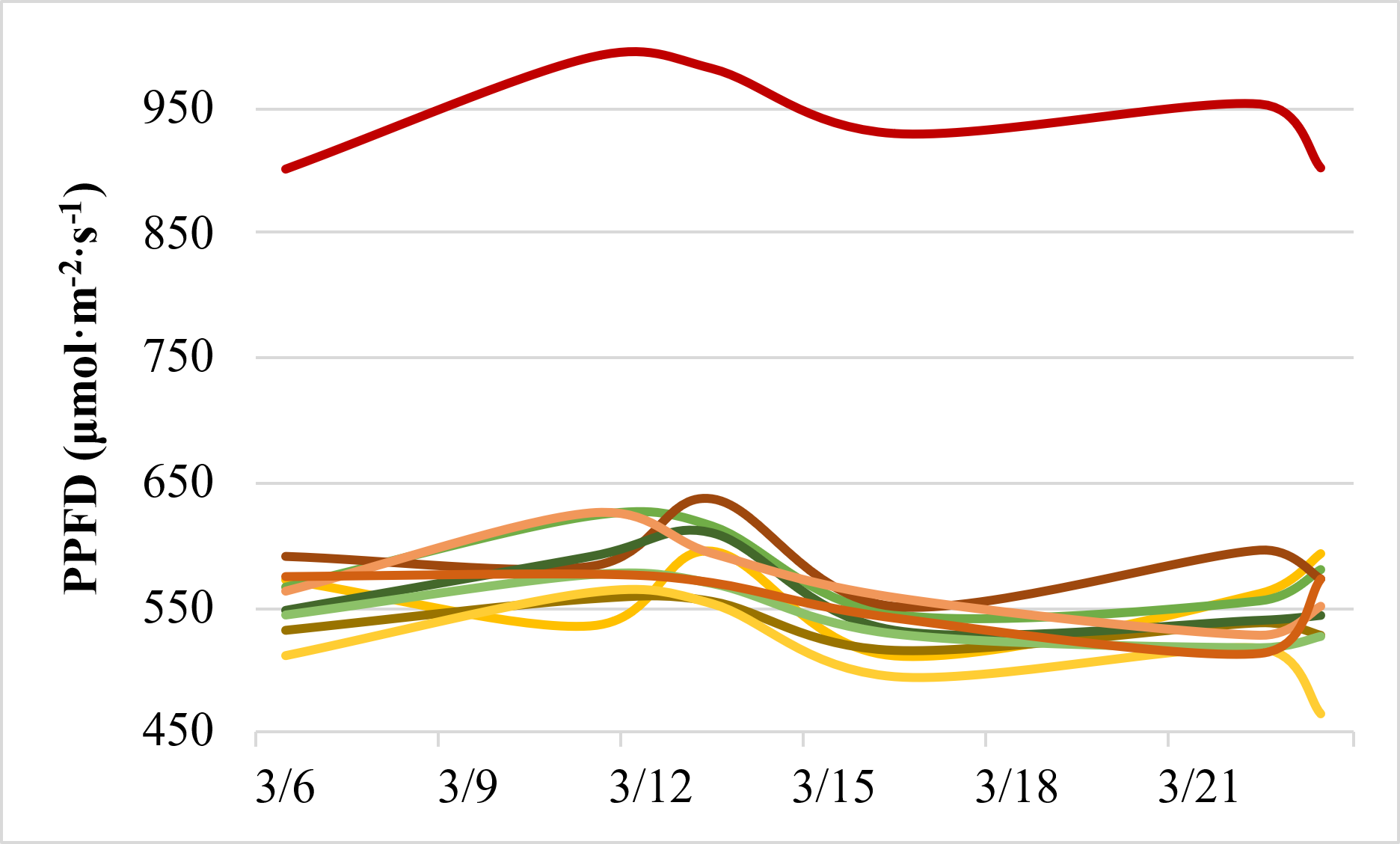


**C**

**D**


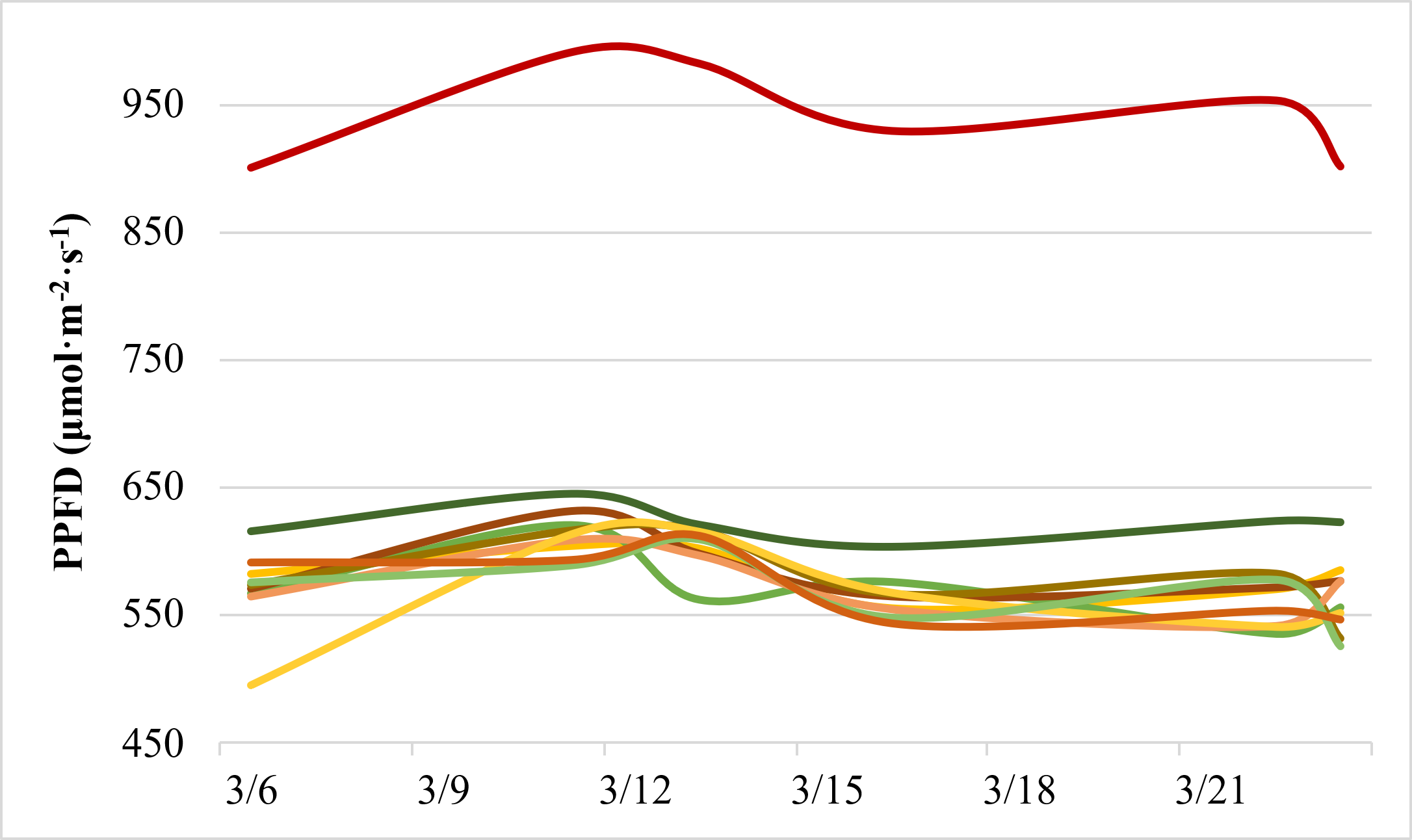

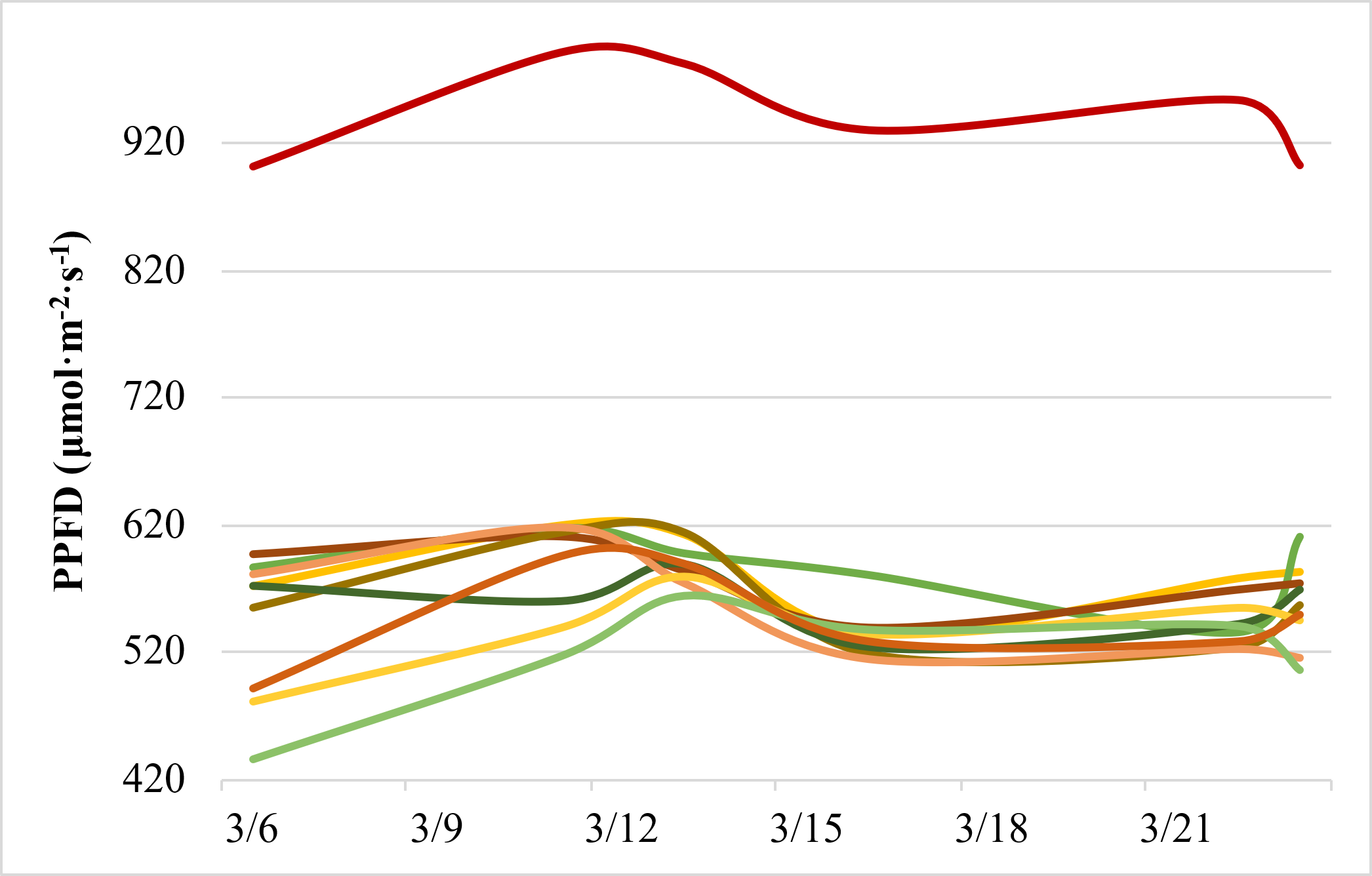


**Supplementary Figure 4.** Irradiance data (global radiation and PAR) **(A)**. Time-based monitoring of light intensity on hydroponic NFT types: modules I **(B)**, II **(C)** and III **(D)**.
